# Experiments on Atmospheric Air and Oxygen Gas

**Published:** 1809-07

**Authors:** 


In one of our former Numbers, we gave an Abstract of the Experiments made at Edinburgh on the changes produced in the atmosphere by respiration and vegetation, and of Dr. Bostock's opinion on the same. The following Experiments, laid before the Royal Society of London, by Messrs. Allen and Pepys, being connected with the question, we have thought it right to select them from the Philosophical Transactions.

1. THAT the quantity of carbonic acid gas emitted is exactly equal, bulk for bulk, to the oxygen consumed; and therefore there is no reason to conjecture, that any water is formed by an union of oxygen and hydrogen in the lungs.

2. Atmospheric air once entering the lungs, returns charged with from 8 to 8½ per cent. carbonic acid gas, and when the contacts are repeated almost as frequently as possible only 10 per cent. is emitted.

3. It appears, that a middle-sized man, aged thirty-eight years, and whose pulse is seventy on an average, gives off 302 cubical inches of carbonic acid gas from his lungs in eleven minutes; and supposing the production uniform for twenty-four hours, the total quantity in that period would be 39,534 cubical inches, weighing 18,683 grains, the carbon in which is 5,363 grains, or rather more than 11 oz. troy: the oxygen consumed in the same time will be equal in volume to the carbonic acid gas. The quantity of carbonic acid gas, emitted in a given time, may depend much on the circumstances under which respiration is performed.

4. When

4. When respiration is attended with distressing circumstances, there is reason to conclude, that a portion of oxygen is absorbed: and as the oxygen decreases in quantity, perception gradually ceases, and we may suppose, that life would be completely extinguished on the total abstraction of oxygen.

5. A larger proportion of carbonic acid gas is formed by the human subject from oxygen, than from atmospheric air.

6. An easy, natural inspiration is from 16 to 17 cubical inches, though this will differ in different subjects; and it is supposed, that the quantity of carbonic acid gas, given off in a perfectly natural respiration, ought to be reckoned at less than at a time when experiments are making on the human subject for the purpose, because in short inspirations the quantity of air, which has reached no farther than the fauces, trachea, &c. bears a much larger proportion to the whole mass required, than when the inspirations are deep.

7. No hydrogen, nor any other gas, appears to be evolved during the process of respiration.

8. The general average of the deficieney in the total amount of common air inspired, appears to be very small, amounting only to 6 parts in 1000.

9. The experiments upon oxygen gas prove, that the quantity of air remaining in the lungs and its appendages is very considerable; and that without a reference to this circumstance, all experiments upon small quantities of gas are liable to inaccuracy.

*The following paper, by the ingenious Mr. Brande, is not less deserving of notice.*

Mr. Brande has laid before the Royal Society, an account of the differences in the structure of calculi, which arise from their being formed in different parts of the urinary passages; and on the effects that are produced upon them by the internal use of solvent medicines. The experiments made by this gentleman were very numerous, and on an uncommonly large collection of calculi, to most of which histories of the case are annexed. The subject is divided into different sections: The 1st. relates to calculi formed in the kidnies, and voided without having undergone any changes in the urinary passages. These are entirely soluble in a solution of pure potash: and when exposed to the action of the blow-pipe, they blacken and emit a strong odour, which arises from the animal matter

 which which they contain, and which occasions the loss in the analysis of these calculi. Its relative quantity is liable to much variation. In one instance a calculous from the kidney, weighing 7 grains, was ascertained to consist of

| | |
|---|---|
| Uric acid . . . . . . . . | 4.5 Grains. |
| Animal matter . . . . . | 2.5 |
| | 7.0 |

In some cases the calculi from the kidnies consist almost wholly of uric acid; sometimes phosphate of lime was combined with the acid.

II. In treating of the calculi which have been retained in the kidnies, and which frequently increase in that situation to a considerable size, he observes that this augmentation is of two kinds.

1. Where there is a great disposition to the formation of uric acid, the calculus consists wholly of that substance and animal matter, so as frequently to form a complete cast of the pelvis of the kidney.

2. Where there is less disposition to form uric acid, the external laminæ are composed of the ammoniaco-magnesian phosphate, and phosphate of lime.

In one instance, a small uric calculus was so deposited on the kidney, that its upper surface was exposed to a continual stream of urine, upon which beautiful crystals of the triple phosphate had been deposited. Mr. Brande therefore infers, that, under common circumstances, a stream of urine passing over a calculus of uric acid, has a tendency to deposit the phosphate upon it.

III. The calculi of the urinary bladder are of four kinds:

1. Those formed upon nuclei of uric acid, from the kidney.

2. Those formed upon nuclei of oxalate of lime from the kidney.

3. Those formed upon sand or animal mucus deposited in the bladder.

4. Those formed upon extraneous bodies introduced into the bladder. These are arranged under the following divisions:—First, Calculi, which from their external appearance consist chiefly of uric acid, and which are mostly or entirely soluble in a solution of potash. Secondly, Calculi composed chiefly of the ammoniaco-magnesian phosphate, or of phosphate of lime, or of mixtures of the two.

two. These are characterised by their whiteness; by exhibiting small prismatic crystals upon their surface, and by their solubility in dilute muriatic acid. Thirdly, Calculi, containing oxalate of lime, commonly called mulberry calculi. These are distinguished by the difficulty with which they are dissolved in acids, by their hardness, and by leaving pure lime, when exposed to the action of the blow-pipe.

By analysis a calculus of 60 grains yielded

| | |
|---|---|
| Urea and muriate of ammonia . . . | 5.2 Grains. |
| Ammoniaco-magnesian phosphate | 6. |
| Uric acid . . . . . . . . . . . . . . . . | 48.8 |
| | 60.0 |

From this and many other experiments Mr. Brande concludes, that the evolution of ammonia depends in all instances upon the decomposition of the ammoniacal salts contained in the calculus, more especially of the ammoniaco-magnesian phosphate, and that no substance which can be called urate of ammonia exists in calculi.

By analysis it was found, that a pure specimen of the mulberry calculus consists of

| | |
|---|---|
| Oxalate of lime . . . . . | 65 Grains. |
| Uric acid . . . . . . . . . . | 16 |
| Phosphate of lime . . . . | 15 |
| Loss in animal matter . . . | 4 |
| | 100 |

IV. The calculi found in the urethra consist of ammoniaco-magnesian phosphate, and phosphate of lime, with a small portion of uric acid; though some appeared to consist almost wholly of ammoniaco-magnesian phosphate.

Mr. Brande, in the next section, has given the result of analysis of the calculi found in the horse, ox, sheep, rhinoceros, dog, hog, and rabbit. These were found mostly to consist of phosphate of lime and carbonate of lime in different proportions. In some, small proportions of animal matter were combined with the other substances.

The inferences drawn from these interesting and important facts are as follow:

That calculi formed in the kidnies, and immediately voided, are almost always composed of uric acid, and that the phosphates are very frequent ingredients in calculi of the bladder. They are uniformly deposited upon extra-

 neous neous substances introduced into the bladder, but never form small kidney calculi. In what is commonly called a fit of the gravel, a small uric calculus is formed in the kidney, and passes along the ureter into the bladder. For, some time after a stone has passed from the kidney, the urine is generally unusually loaded with uric acid, and deposits that substance upon the nucleus now in the bladder. After this, the subsequent additions to the calculus consist principally of the phosphates.

Where the disposition to form uric acid in the kidneys is very great and permanent, the calculus found in the bladder is principally composed of uric acid; but where this disposition is weak, the nucleus only is uric acid, and the bulk of the stone is composed of the phosphates. When the increased secretion of uric acid returns at intervals, the calculus is composed of alternate layers of uric acid and the phosphates. There are besides these many variations in the formation of the calculi.

In speaking of the solvents, Mr. Brande admits, that the internal exhibition of the alkalies often prevents the formation of the uric acid, and of course an increase of a calculus in the bladder, as far as the uric acid is concerned; but that its action will not proceed any farther; because from his experiments he finds there is at all times a quantity of uncombined acid in the urine; and hence it follows, that, although the alkali may arrive at the kidneys in its pure state, it will there unite with the uncombined acid, and be rendered incapable of exerting any action upon the calculus in the bladder. Mr. B. also observes, that whenever the urine is deprived of a portion of the acid which is natural to it, the deposition of the triple phosphate and phosphate of lime more readily takes place, which is effected by the exhibition of the alkalies; and, therefore, though alkaline medicines often tend to diminish the quantity of uric acid, and thus prevent the addition of that substance in its pure state to a calculus in the bladder, they favour the deposition of the phosphates.

With regard to the exhibition of the acids, particularly the muriatic acid, in order to dissolve the phosphates, Mr. B. admits, that, during the use of this acid, the phosphates are either diminished, or disappear altogether; and even the urine acquires sometimes an additional acidity, and therefore a solution of that part of the calculus, which consists of the phosphates, may be expected; but even then the nucleus of uric acid would remain, and thus a great great deal of time would be lost without any permanent advantage. He is also decidedly against the injection of these solvents into the bladder, at once, by means of instruments; because, in every case that has come under his observation, it has always aggravated the sufferings of the patient. He concludes, that as the nuclei of calculi originate in the kidnies, and that of these the greater number consist of uric acid; the good effects so frequently observed during the use of an alkali, arise not from any actual solution of calculous matter, but from the power which it possesses of diminishing the secretion of uric acid, and thus preventing the enlargement of the calculus; so that, while of a very small form, it may be voided by the urethra.

